# 

**Published:** 1907-04-15

**Authors:** 


					﻿Dental
Register
NELVIULE S. HOFF, Editor,
______!	Ann Arbor, Mich.
Vol. LXI----APRIL, 1907---- No. 4
SAMUEL A. CROCKER & CO., Publishers
OHIO DENTAL AND SURGICAL DEPOT
35 W. FIFTH STREET, CINCINNATI, 0.
PRICE, ONE DOLLAR A YEAR
Entered at the Postoffice at Cincinnati, O., as second-class mail matter.
				

